# Gender disparity in survival of early porcine fetuses due to altered androgen receptor or associated U2 spliceosome component

**DOI:** 10.1038/s41598-023-41665-6

**Published:** 2023-09-12

**Authors:** Kelly Zacanti, Insung Park, Bret R. McNabb, Tara Marie Urbano, Elizabeth A. Maga, Barbara Jean Nitta-Oda, Joan D. Rowe, Sadie L. Hennig, Pablo Ross, Trish Berger

**Affiliations:** 1 Department of Animal Science, University of California Davis, Davis, CA USA; 2 Department of Population Health and Reproduction, University of California Davis, Davis, CA USA

**Keywords:** Developmental biology, Physiology, Endocrinology

## Abstract

A single locus on the X chromosome codes for androgen receptor (*AR*) although this gene is subject to alternative splicing. *AR* is expressed in multiple tissues in males and females and is essential for reproductive success in the male. Since male and female mice are viable following naturally occurring and engineered loss of function with male mice infertile as anticipated, functional deletion of *AR* in pigs was hypothesized to provide a genetic containment strategy for males with edited genomes. In addition, deletion of *AR* might be a method to manage boar taint, hence contributing to a perceived improvement in animal welfare. The CRISPR/Cas9 technology was used to edit either exon 2 or exon 5 of the pig *AR* gene*.* Although pregnancies were established following embryo transfer of edited embryos, they were not maintained beyond day 25. Furthermore, normal M:F sex ratios were present in edited blastocysts and 19-day fetuses, but all fetuses recovered on day 21 or later were female. The pig *AR* gene differs from the mouse in having a U2 spliceosome component encoded in the intronic region. Hence, the absence of fetal survival beyond day 25 may be due to interference with the *U2* component rather than *AR*.

## Introduction

The use of gene editing and genetic engineering in livestock has the potential to improve disease resistance, productivity and sustainability of animal agriculture. However, concerns remain about the safety of animals produced by these technologies. These include the specificity of gene editing events and containment of genetically modified animals to prevent introduction of an induced modification into wild-type animals. Transgene containment strategies in animals include standard species-specific reproductive practices (for instance castration) or more recently, ablation of germ cells during early embryo development^1^, both intended to produce sterile animals. Another potential approach for biocontainment of genetically modified food animals is to eliminate the function of the androgen receptor via gene editing to concomitantly reduce androgen production in the testis and thus fertility. This approach has the added benefit of preventing the formation of androstenone, a compound formed from the metabolism of androgens that is implicated in “boar taint” or the stale urine and fecal fragrances that accumulate in the meat of male pigs as the animals pass into puberty^2–6^. Sterile males would reduce the potential for transfer of a transgene beyond breeding stocks and elimination of androstenone would alleviate the need for castration of male pigs thus improving the welfare, efficiency and sustainability of pig production.

Androgen receptor (*AR*) is transcribed and translated from a single gene on the X-chromosome. The primary function is as a nuclear transcription factor although alternative transcripts exist and fast, nongenomic signaling occurs^7–9^. The importance of *AR* in development and function of the male reproductive system is widely accepted. Decades ago, this importance was demonstrated in male mice lacking a functional AR^10^. Although AR is clearly expressed in female reproductive tissues, a functional AR in tissues is not necessary for fertility in female mice nor for postnatal survival of male mice^11,12^. A component of the U2 spliceosome^13,14^ is located within the intronic sequence of the porcine *AR* but not within the intronic sequence of the murine *AR*^13,14^. Spliceosomes bind to intronic sequence prior to pre-mRNA splicing with their function being critical in gene function including altered mRNA splicing during embryo/fetal development^15–19^.

This demonstrated compatibility of AR-deficit with survival, male infertility, and female fertility led to the hypothesis that a CRISPR-induced deletion^20,21^ in *AR* would provide a male model compatible with additional gene editing of males that would allow inherent genetic containment of generated research animals. In addition, deletion of functional AR in male pigs might eliminate boar taint in uncastrated males, providing either a direct solution to a perceived animal welfare issue or providing a path for further research. Since the *AR* gene is located on the X chromosome and hence not inherited from the sire, all male offspring from homozygous *AR* deficient females bred to wild type males would lack a functional AR.

Our initial goal was to generate *AR*-edited piglets to further these studies on genetic containment and elimination of boar taint. Although initial results indicated that *AR*-edited male blastocysts were present at the anticipated male:female ratio and *AR*-edited male fetuses were present 19 days after fertilization, only female fetuses were present 21–25 days post fertilization and typically no edited fetuses survived for 25 days or longer. Subsequent goals included evaluation of expression of *AR* before gonadal sex differentiation^22–24^; of conceptus 17 alpha hydroxylase (*CYP17A1*) as rate limiting enzyme in synthesis of androgens, the AR ligand; and of the U2 spliceosome component located in the intronic region of the porcine AR.

## Results

### Efficacy of selected CRISPR guide RNAs

Initially, guide RNA (gRNA) and Cas9 enzyme were microinjected into in vivo produced zygotes as an established technique until electroporation of gRNA and Cas9 into in vitro produced zygotes was optimized and became standard practice in our laboratories. The first experiments with each technique were to culture resulting embryos in vitro to verify each of the three CRISPR gRNAs (Table 1) efficiently mutated AR sequence in the translated region and were compatible with development to blastocyst (greater than 10% developing to blastocyst). Mutation rates (combined for microinjection and electroporation) for individual gRNAs ranged from 65 to 100%.

**Table 1:** The guide RNA sequences designed to alter the *AR* sequence and efficacy of editing.

Guide	Guide RNA sequence (5′ to 3′)	Exon	Mutation rate^a^ after microinjection	Mutation rate^a^ after electroporation
AR_2A	GCACCTCGAAAGGTCTTGGA	2	3/3 (100%)	12/15 (80%)
AR_2B	GCTCTCCGGGTGGCACTCAG	2	4/4 (100%)	8/8 (100%)
AR_2A + AR_2B	GCACCTCGAAAGGTCTTGGA & GCTCTCCGGGTGGCACTCAG	2	–	15/18 (83%)
AR_5	ATGTGACACTGTCAGCTTCT	5	4/7 (57%)	9/13 (69%)

^a^Mutation rate was calculated as proportion of blastocysts with indels after microinjection of in vivo produced zygotes or electroporation of in vitro produced zygotes.

### Pregnancy in recipients of embryos with edited AR sequence

**Figure 1:**
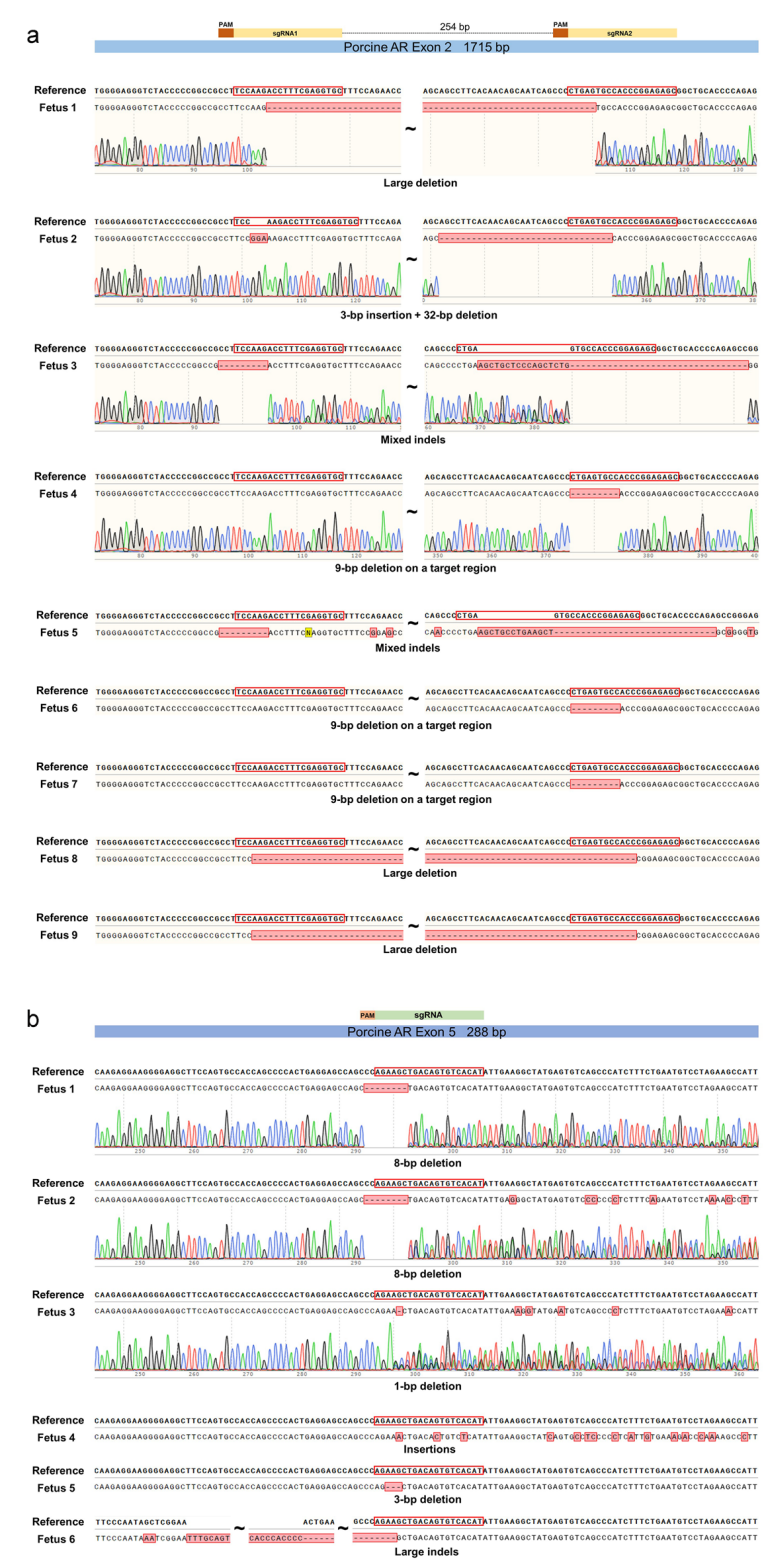
Sequences of 22–25 d fetuses edited at Exon 2 (**a**) or Exon 5 (**b**). Top sequence for each sample shows the reference sequence with target region(s) outlined in red in the initial part of the translated region. The lower sequence for each sample indicates the actual sequence for that fetus with sequencing traces included for some fetuses in each group. (**a**) As indicated by the two outlined target regions, fertilized zygotes were electroporated with a combination of two guide RNAs targeting Exon 2. (**b**) An additional fetus with some mosaicism is not included.

After verification that microinjected gRNAs efficiently edited the zygotes and were compatible with development of edited embryos to blastocysts, edited zygotes (in vivo fertilized) were transferred to two recipients (weaned sows). One recipient returned to estrus and aborted conceptus tissue was detected in the pen of the second on day 26. Recipients (n = 3) receiving contemporaneous transfers of embryos with different gene edits were pregnant on days 28–30 by ultrasound diagnosis although pregnancies were lost prior to delivery of live offspring. Subsequent transfers were of embryos developing following in vitro maturation of oocytes and in vitro fertilization; transfers of such embryos with edits targeting other genes were compatible with maintenance of pregnancy to at least day 45 (ultrasound) and presence of normal fetuses at day 49. In 11 recipients with anticipated pregnancy with fetuses containing CRISPR-edited *AR*, 10 exhibited indications of initial establishment of pregnancy including absence of return to estrus. Aborted conceptus tissue was detected in a pen of an individually housed recipient between day 26 and day 28. Subsequently, four of the recipients were examined after euthanasia on day 25; ovaries displayed a transition from corpora lutea to large corpora albicans consistent with initial pregnancy and a delayed return to estrus. Fetuses were obtained from the five remaining recipients of *AR* edited embryos, two recipients at 22 days post-ovulation, two at 23 days post-ovulation and one at 25 days post-ovulation. All fetuses described as edited had an altered DNA sequence confirmed by Sanger sequencing. Nine of the recovered 22–25 day edited fetuses had 1–283 bp deletions that would induce frame shift mutations in large regions of translated AR protein. Five more of the recovered fetuses had bp deletions that would eliminate 92, 24, 3, 3, or 1 amino acid from translated protein. Another fetus had 15 bp indels that would cause 11 amino acid substitutions in a translated protein. The single edited fetus characterized only by mosaicism may have had normal AR function in most cells. Specific edits of these fetuses are detailed in Fig. 1 with edits being almost exclusively biallelic.

**Figure 2:**
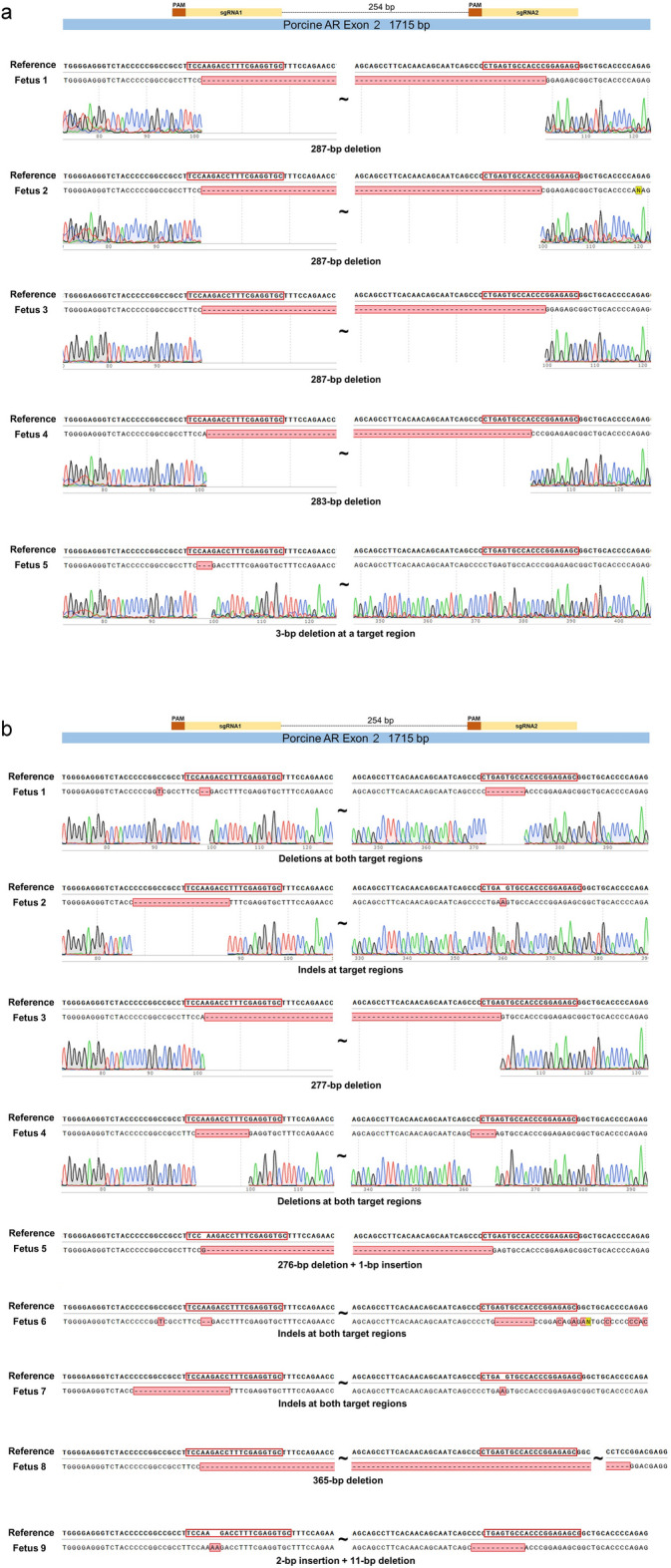
Sequences of (**a**) 19 d fetuses and (**b**) 21 d fetuses edited at Exon 2. The two targeted regions are outlined in red near the beginning of the translated region in the reference sequence at the top. The lower sequence indicates the actual sequence for each fetus with sequencing traces included for some fetuses. An additional fetus with some mosaicism is not included in (**a**).

In a follow-up experiment, both recipients receiving embryos electroporated with Cas9/gRNAs targeting the *AR* gene were pregnant with edited fetuses with one recipient evaluated at 19 days post-ovulation and in the other recipient evaluated at 21 days post-ovulation. Again, the vast majority of fetuses had 10 to 365 bp deletions and occasional small insertions that would alter a large region of a translated AR protein. One fetus had a 3 bp deletion that would eliminate a single amino acid from the translated protein and the mosaic fetus may have had normal AR function in most cells. Altered sequences of edited fetuses are reported in Fig. 2.

### Gender and gene expression

**Figure 3:**
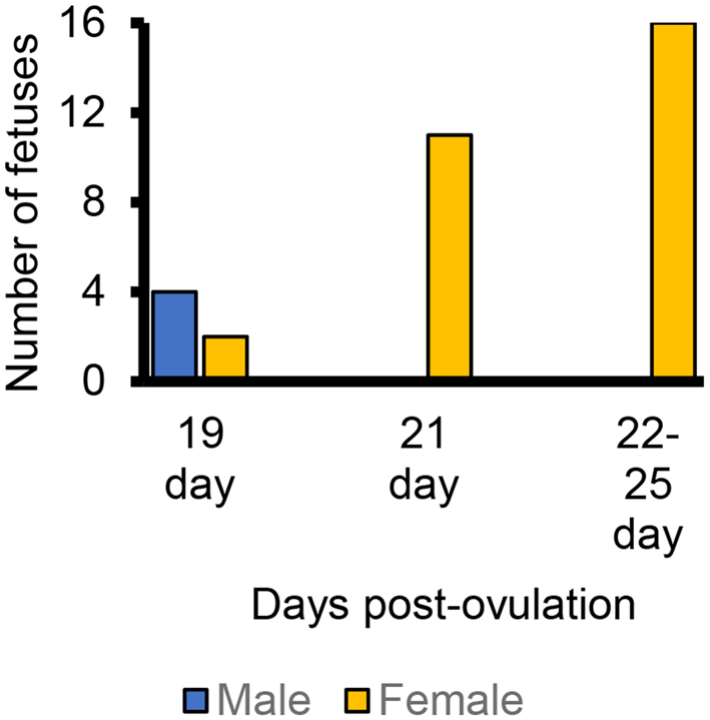
Gender of recovered fetuses with edited *AR* sequence. Male:female ratio in fetuses older than 20 days differs from expected 1:1 ratio (P < 0.001 by chi square analysis).

Gender determined in each of the fetuses recovered from a recipient using PCR primers specific for X and Y chromosomes demonstrated all of the 21 day and older *AR*-edited fetuses were female (Fig. 3). This sex ratio was skewed from the anticipated 1:1 sex ratio (P < 0.001). Male fetuses (edited) were present at 19 days and gender analysis of blastocysts developed from *AR*-edited zygotes indicated no bias for female embryos had developed prior to the blastocyst stage (7:8 male:female in randomly selected group of *AR*-edited blastocysts with equivalent male:female ratios regardless of exon targeted).

**Figure 4:**
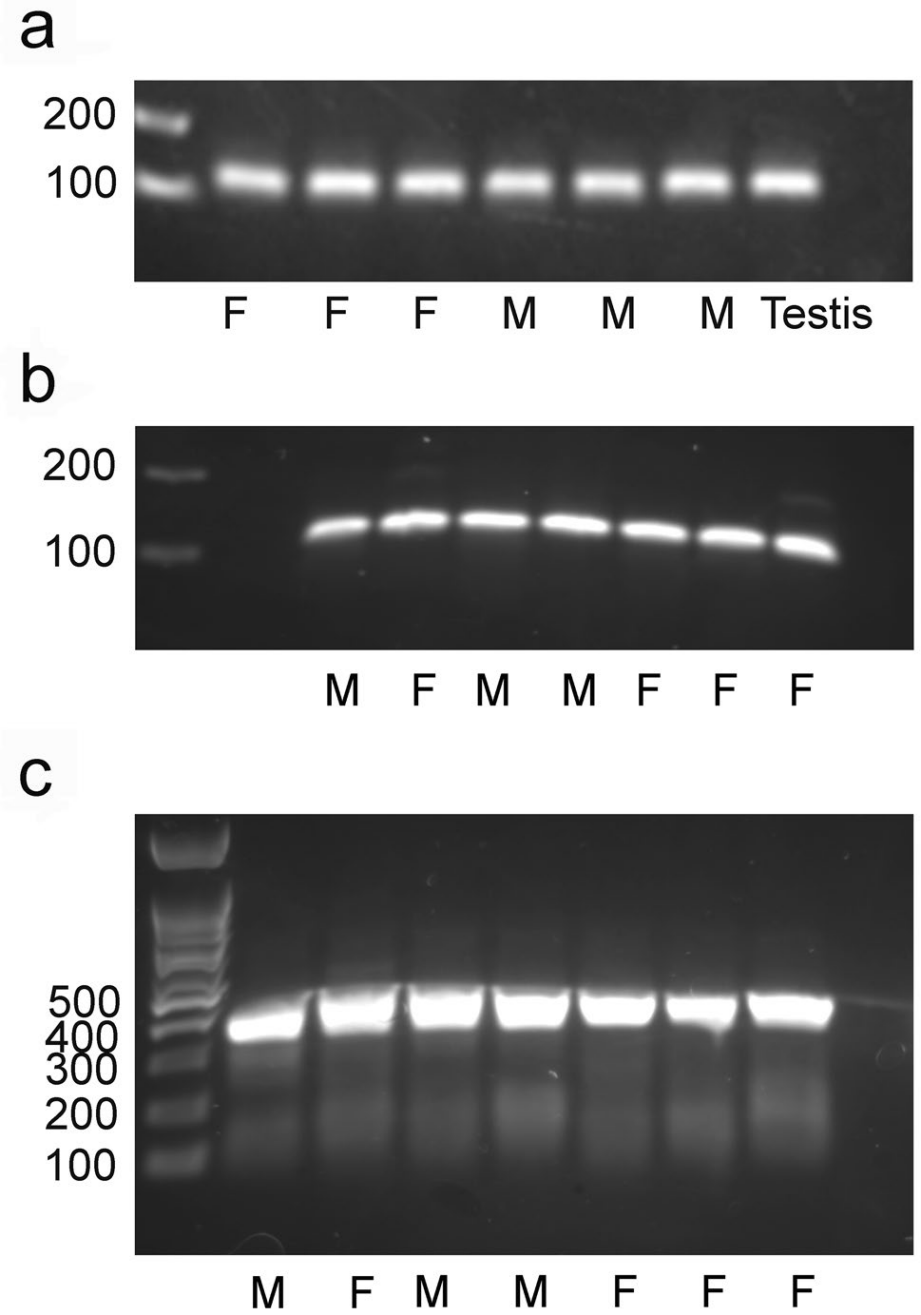
*AR* transcript is present in unedited male and female 19-day fetuses. (**a**) Presence of *AR* transcript in whole fetuses produced by in vivo fertilization) and assessed with primers to detect 108 bp product with a positive control, the testis, on the right. (**b**) Presence of *AR* transcript (108 bp product) in the heads of additional 19-day fetuses with one male and one female fetus being electroporated fetuses that were not successfully edited and the remaining four fetuses being unmanipulated fetuses. (**c**) Presence of *AR* transcript (357 bp product) in the heads of the same 19-day fetuses examined in (**b**). The bp in the ladder are indicated to the left of the blot and gender (female (F) and male (M)) is indicated below the blot. (Images of the entire blots are visible in Supplementary Figure 1).

Evaluation of mRNA isolated from entire 19 day wild-type fetuses demonstrated that *AR* was expressed in male and female fetuses (Fig. 4a) with no obvious difference between male and female fetuses. Isolation of mRNA from heads of additional 19-day fetuses indicated *AR* expression occurred in the head of 19-day fetuses (Fig. 4b and c) as well as the body. A qPCR analysis also indicated transcripts of *AR* were detectable but at low levels in heads and no difference in expression was detected between male and female fetuses (*AR*∆Cts of 10.8 vs 11.7, SE 0.4 for control male and control female fetuses, respectively; P = 0.17).

**Figure 5:**
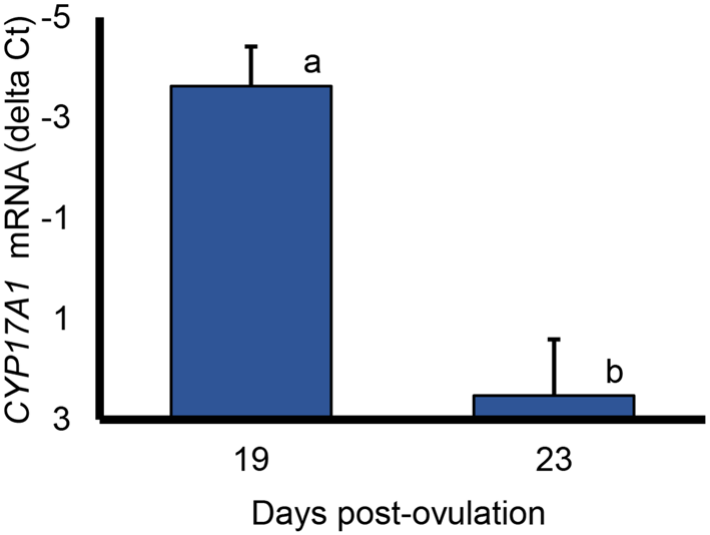
Expression of *CYP17A1*, a rate limiting enzyme in androgen synthesis, in porcine placenta. Values represent the means (and SEM) in placentas from four 19-day fetuses and two 23-day fetuses. a,b indicates means differ, P = 0.01. *CYP17A1* was not detectable in placentas from three 48-day fetuses.

Expression of the rate-limiting enzyme in testosterone synthesis *CYP17A1* was verified in the placentas of 19 day control pig fetuses, was decreased in placentas at 23 days post fertilization (Fig. 5; P = 0.01) and was no longer detected in placentas from day 48 and day 54 fetuses. The level of *CYP17A1* expression was similar in placentas at 19 and 23 days post fertilization and pre- and postpubertal testes (ΔCt of − 1.58 ± 1.2 vs − 3.96 ± 2.25, P > 0.25). Expression of *CYP17A1* was quite low in fetuses in the 19 to 23-day range (more than 250-fold lower than placentas; ΔCt of − 4.5 ± 1.0 vs 7.0 ± 1.0 for placentas and fetuses, respectively, P < 0.001) without a clear difference between male and female fetuses.

### Potential response to AR editing

Transcripts of the *U2.35* spliceosome located within the intronic region of *AR* between exons 3 and 4 were assessed by qPCR. Although transcript levels were quite low, they were detectable in control fetuses 19 days post fertilization but were further reduced in the two AR-edited male fetuses assessed 19 days post fertilization (U2.35 ΔCt of 16.0 ± 0.4 vs 19.5 ± 0.5; P = 0.01 for control and *AR*-edited fetuses). The U2.35 transcript levels were reduced in these *AR*-edited males regardless of whether the comparison was with control male fetuses or control male and female fetuses. In contrast, transcript level of this spliceosome did not appear to be affected in edited female fetuses at either 21 or 23 days post fertilization (U2.35 ΔCt of 15.4–16.9 in 19–23 d control female fetuses vs 15.4–17.4 in *AR*-edited 21–23 d edited female fetuses, SEM = 0.6–0.8; P > 0.5).

## Discussion

### Editing efficiency

All three of the *AR* gRNAs tested were effective with edits observed in a high proportion of both blastocysts and recovered fetuses. Exon 2 contributes to all three of the AR transcripts described in *Sus scrofa*, hence edits in exon 2 would affect all three transcripts. In contrast, exon 5 is not present in the smallest transcript and was chosen as the target for one of the gRNAs in case the resulting edited fetuses had a less severe phenotype. The 1–365 bp edits (primarily deletions) in the translated regions would impact amino acid sequence and protein confirmation of translated protein but directly assessing functional significance requires knowledge of physiological function, currently an unknown for AR in 19-day pig fetuses. Evaluating the surviving edits in fetuses recovered early in gestation may be desirable to further evaluate potential guides and targets in future CRISPR editing of other genes. Although the embryos transferred to four of the five recipients maintained to 22–23 days post fertilization were a mixture of potential edits targeting the two different exons, editing efficiency after electroporation was slightly lower in the blastocysts exposed to the gRNA targeting exon 5. Hence, the tendency for fetuses with edits targeting exon 5 to be underrepresented (P = 0.07) compared with the ratio of transferred embryos electroporated with gRNAs targeting the two exons may be partially due to this slightly lower editing efficiency rather than a potential effect on fetal viability.

### Species differences in survival of AR deficient fetuses

The inability of edited *AR* pig fetuses to survive to 4 weeks of age was surprising. In contemporary transfers differing only by target gene, pregnancy was maintained past this timepoint. Although four fetuses are required to maintain porcine pregnancy beyond 3 ½ weeks of gestation^25,26^, four of the five transfers examined between days 22 and 25 had at least four fetuses. Therefore, it seems unlikely that the pregnancies from transfer of *AR*-edited embryos lost shortly after day 23 would consistently have less than four fetuses, suggesting that the *AR*-edited female fetuses were also of limited viability. Multiple transfers of female embryos with *AR* edits mixed with other edited embryos capable of longer embryonic survival is a means to more conclusively evaluate hypothesized, limited viability. Off-target edits of essential developmental genes might be another potential explanation, but evaluation of the most likely off target edits for these gRNAs did not identify any such edits in these fetuses (Pinho, personal communication). Although expression of AR is necessary for male reproductive competence, minimal expression (Tfm) or deletion are compatible with survival of mice to adults^12,27–29^. Expression of *AR* in control pig fetuses at 19 days post fertilization indicated AR might have a role in development although no differences were noted between male and female fetuses, consistent with initial sexual differentiation a week later^30,31^. The enzyme CYP17A1 is a/the rate limiting enzyme in testosterone synthesis. Transcript presence in the placenta from these very young conceptuses prior to expression in the fetus suggests the placenta serves as an initial, transient source of androgen to bind to androgen receptor in the fetus, although transcript decreases rapidly and was not detectable by day 48 of pregnancy. Placental *CYP17A1* transcript abundance in the early placenta was similar to that in postnatal testes consistent with the placenta as a source of androgen during early development. Although detectable, fetal transcript abundance was less than 1% of that in the placenta suggesting the fetus itself was a less likely source of androgen. The potential presence of androgen and the androgen receptor a week before sex differentiation suggested androgen had an additional role in early development. Similar to mouse embryos, presence of AR transcripts was verified in the head of the early pig embryos in these studies. Altering AR transcript level in the conceptus head might contribute to loss of fetal viability in these early pig fetuses but survival of *AR* edited mouse fetuses indicates that functional *AR* transcripts in the brain are not required for mouse survival.

### Early loss of AR-edited male fetuses

The loss of *AR*-edited male fetuses observed in this study between 19 and 21 days raises the issue of the physiological basis for the sex-influenced initial loss. Since the *AR* gene is located on the X chromosome, males have one copy and females have two copies. Hence, edited female fetuses might be monoallelic or biallelic while male fetuses are either edited or not edited (if we ignore potential minor mosaicism). If the 22 day or older female fetuses were monoallelic, the presence of an AR in some of the fetal cells might explain the prolonged survival of female fetuses compared with the absence of male fetuses from 21 days post fertilization. However, since only one of the females was monoallelic, the presence of an unedited *AR* in some of the fetal cells is not an adequate explanation for the prolonged survival of the female fetuses.

Although fetal male mice were believed to have higher *AR* levels in the brain than female fetuses at day 16 of gestation^32^ or similar levels at day 17 in the hippocampus and hypothalamus but higher levels at postnatal day 1^33^, no difference in *AR* transcript levels in the head were detected in male and female control fetuses at day 19 post fertilization in the current study. Hence, if *AR* expression is similar in male and female fetuses, editing *AR* leading to reduced transcript levels is unlikely to explain the earlier loss of viability of male fetuses between days 19 and 21.

The current Ensembl *Sus scrofa* genome build indicates that one of the U2 spliceosomes is located in the intronic region of *AR* while the murine *AR* does not contain such a spliceosome in the intronic region. Hence, disruption of the *AR* in *Sus scrofa* could lead to reduced transcripts for the U2 spliceosome. Although many paralogues of the U2 spliceosome exist, the one located in the intronic region of the *AR* has no more than 66.1% identity to any of the other U2 spliceosomes. The current thought that some of the genes on the X-chromosome partially escape X-chromosome inactivation^34,35^ in conjunction with partial rather than complete reduction of transcription with edits of the *AR*, might explain the observed sex bias. If this were the case, reduced expression of this U2 spliceosome would be expected in the 19-day male fetuses with edited *AR* compared with control male (and female) fetuses as we observed. A limitation of this work is that expression of the U2 spliceosome is very low, particularly in edited male fetuses, higher than water blanks but not at a level that can be confidently quantified. We did not see a later reduction in edited female fetuses suggesting that their later loss was due to an alternative mechanism or a relative reduction compared with control female fetuses that occurred slightly later in development.

In conclusion, CRISPR editing of the *AR* gene led to a sex biased loss of male fetuses between day 19 and 21 and subsequent loss of female fetuses. Further work is required to determine if editing *AR* in general is embryonic lethal in pigs and if this is a viable approach to produce sterile males for transgene containment and to reduce boar taint.

## Methods

### Animals and overview of experiments

All experiments were approved by and performed in accordance with the Institutional Animal Care and Use Committee (IACUC) at the University of California, Davis (#18549, #20394, and #22434) consistent with the Guide for the Care and Use of Agricultural Animals in Research and Teaching and ARRIVE guidelines. All animals were housed at the UC Davis Swine Facility.

### Retrieval of in vivo fertilized zygotes and microinjection

Peripuberal gilts (100 kg BW) were injected with PG 600^®^ (400 IU pregnant mare’s serum gonadotropin and 200 IU human chorionic gonadotropin per dose, Merck, Kenilworth, NJ) followed by Chorulon^®^ (750 IU human chorionic gonadotropin per dose, Merck) to induce timed ovulation. Females were inseminated 30 h after Chorulon^®^ injection and embryos recovered 48 h after Chorulon^®^ injection. Zygotes were microinjected with Cas9 protein and gRNA or combination of gRNAs via cytoplasmic microinjection 9 to 10 h post-fertilization. Any embryos that divided before microinjection were discarded. Guide RNAs were diluted to a final concentration of 25 ng/μL and the concentration of the Cas9 protein was 50 ng/μL. When a combination of gRNAs was microinjected into zygotes, their combined final concentration was also 25 ng/μL.

### In vitro production of embryos

Pig ovaries were collected from a slaughterhouse (Olson Meat Company, Orland, CA) and transported to the laboratory in a thermos with saline (35 °C). Oocytes were aspirated from medium sized follicles (3–5 mm) with a 20-gauge needle attached to a 10 mL syringe. Collected follicular fluid was transferred to a Petri dish and searched for oocytes. Oocytes were washed in maturation medium (TCM-199 (Gibco M2154, ThermoFisher) supplemented with 10% porcine follicular fluid, 50 μg/mL gentamicin, 100 μg/mL cysteine, 0.91 mM sodium pyruvate, 3.05 mM d-glucose, 0.5 mg/mL FSH, 0.5 mg/mL LH, 100 ng/mL EGF, and 1 mM dibutyrl cAMP)^36^, placed in a final drop of maturation medium covered in mineral oil, and placed in an incubator (38.5 °C, 5% CO_2_, 6.7% O_2_, approximately 90% humidity) for 20 to 22 h. Oocytes were then moved to a new plate containing maturation medium (TCM-199 supplemented with 10% porcine follicular fluid, 50 μg/mL gentamicin, 100 μg/mL cysteine, 0.91 mM sodium pyruvate, and 3.05 mM d-glucose) for an additional 20 to 22 h of maturation. Oocytes were matured for a total of 40 to 44 h to the MII stage. Once matured to the MII stage, oocytes were stripped of their cumulus cells using 1% hyaluronidase (1 mg/mL) in TCM-HEPES (Sigma) and washed in modified Tris-Buffered Medium (mTBM) (sterile ultrapure water containing 113.1 mM NaCl, 3 mM KCl, 7.5 mM CaCl_2_⋅2H_2_0, 20 mM Tris Base, 11 mM d-glucose, 5 mM sodium pyruvate, 2 mg/mL bovine serum albumin, 1 mM caffeine, pH 7.3–7.4 after equilibration, and phenol red^36,37^). Five 90 μL drops of TBM medium were placed in each 60 mm Petri dish. Groups of 20 oocytes were pipetted into the 90 μL drops of TBM and plates were placed into an incubator (38.5 °C, 5% CO_2_, 6.7% O_2_, approximately 90% humidity).

Fresh semen was collected from boars housed at the UC Davis Swine Facility and transferred to the laboratory in a concealed 50 mL conical tube. In the laboratory, semen was kept warm on a heat block (38.5 °C). Sperm motility was assessed by viewing a 5 μL drop of raw semen on a slide under a compound microscope. Semen (100 μL) was pipetted into a 15 mL conical tube containing 1.9 mL of TBM medium and the sperm were washed by centrifugation (120×*g*) for 3 min. Supernatant was aspirated and discarded. TBM (1.9 mL) was added to the sperm pellet and gently pipetted to mix. The sperm were washed by centrifugation (100×*g* for 3 min). Supernatant was aspirated and discarded. TBM (500 μL) was added to resuspend the pellet. Sperm motility was assessed, as previously stated. Sperm were counted using a hemocytometer. Oocytes were fertilized by adding 10 μL of sperm prepared in TBM medium with the concentration adjusted to 1000 sperm per oocyte (20,000 sperm per 10 μL) and gametes co-incubated. Presumptive zygotes were washed in porcine zygote medium^38^ (108 mM NaCl, 25.07 mM NaHCO_3_, 10 mM KCl, 0.35 mM KH_2_PO_4_, 0.4 mM MgSO_4_⋅7H_2_O, 2 mM Ca(lactate)_2_⋅5H_2_O, 0.2 mM sodium pyruvate, 2 mM l-glutamine, 5 mM hypotaurine, 2% MEM Essential Amino Acids (50x), 1% MEM Non-Essential Amino Acids (100×), 10 μg/mL gentamicin, 3 mg/mL BSA), moved to a 4-well plate containing this same medium and incubated.

### CRISPR guide RNAs and primers

Three gRNAs were designed using a CRISPR design tool from the Zheng lab at MIT (crispr.mit.edu) to target exons 2 and 5 of *AR* in the *Sus Scrofa* genome 11.1 (Table 1). Targets were chosen in translated regions near the start of the exon. Exon boundaries were determined with ENSEMBL release 109^39^. The CRISPRevolution sgRNA EZ Kits (Synthego, Menlo Park, CA, USA) were purchased for each selected gRNA. Primers were designed^40^, to amplify regions of the *AR* gene containing each gRNA target site (Table 2).

**Table 2:** PCR primers used to amplify DNA.

Primer name	Product size (bp)	Target	Sequence ID	Sequence (5′→3′)
AR_610F AR_610R	610	*AR*_5 Exon 5	NC_010461.5	TCAGGGAGAAAGCAGGATACG AGGGCAAGAAAGGAACAGACAT
AR_792F AR_792R	792	*AR*_2A and *AR*_2B Exon 2	NC_010461.5	ACGCATCGTAGCCTGTTGAA TGGACACCGACACTGCCTTA
AR_1170F AR_1170R	1170	*AR*_2A and *AR*_2B Exon 2	NC_010461.5	CATCCCTCTCTGCTTGCTGAA CTGACTATGTCTGCGGGTTGA

### Electroporation of in-vitro produced zygotes

Nine hours post-insemination, 35 to 50 embryos were moved into SOF-HEPES followed by washing through three drops of Opti-MEM™ (Gibco). The embryos were added to a drop containing 10 μL Opti-MEM™, 5 μL Cas9 protein (200 ng/μL), and 5 μL gRNA (100 ng/μL) or combination of gRNAs (100 ng/μL). This 20 μL drop containing embryos was pipetted into a 1 mm gap cuvette (BioRad, Cat No. 1652083). Embryos were electroporated with 5 bipolar 30 V pulses, 1 ms each with a 100 ms pulse width and 0% decay using a NEPA21 Type II super Electroporator (NEPAGENE, Ichikawa, Japan). After electroporation, embryos were moved to porcine zygote medium and incubated.

### Embryo transfer

Recipients were synchronized with the goal of recipients of in vivo fertilized zygotes ovulating half a day after fertilization of the oocytes and recipients of in vitro fertilized embryos ovulating a day after fertilization of the oocytes. Females serving as recipients of the in vivo generated zygotes were weaned, injected with PG600 followed 3 days later with Chorulon^®^. Recipients of in vitro generated zygotes were synchronized with a combination of Matrix (Animal Health International, Ceres, CA, USA), PG600, and Chorulon^®^ with some gilts sampled 19–23 days post fertilization not receiving Matrix as part of their synchronization.

Anesthesia was induced in recipients with an intramuscular injection of Telazol^®^ (Zoetis, Parsippany, NJ) followed by isoflurane via inhalation. When the level of anesthesia was adequate for intubation, the recipients were intubated and anesthesia was maintained with isoflurane. Ovaries and oviducts were accessed through an approximate 8 cm midventral incision. After inspection of ovaries to visually verify appropriately timed ovulation, 2 to 4 cell embryos (64–80) were transferred into oviduct ampulla from the cranial end with approximately 100 µL of porcine zygote medium.

### Evaluation of pregnancy and recovery of fetuses

For 3 days beginning 19 days after ovulation, recipients were evaluated for return to estrus. High fecal progesterone determined in supernatant from a 10% fecal water homogenate further confirmed establishment of pregnancy (Rapid Bovine Progesterone Kit, Biometallics, Inc, Princeton, NJ, USA). Although a skilled operator was able to detect pregnancy in day 28 pregnant sows with ultrasound, no recipients were observed pregnant at day 28 following transfer of edited *AR* embryos. Subsequently, recipient females were euthanized at days 19–26 and the reproductive tracts were removed and transferred (< 5 min transfer time) to the laboratory. Ovaries were examined for presence of corpora lutea (CLs), presence of large, sometimes slightly pink corpora albicans indicative of CL regression in last day, and potentially size of largest antral follicles. Each uterine horn was cut open and fetuses were recovered. Fetuses were quickly examined prior to either flash freezing in liquid nitrogen if RNA was to be evaluated or freezing at – 17 °C when only DNA evaluation was planned. In some cases, extraembryonic tissue was flash frozen in liquid nitrogen and the amnion was separated from the chorioallantois before freezing of extraembryonic tissues from 49-day control fetuses. Sometimes, the head of the fetus was separated from the remainder of the body prior to flash freezing.

### Evaluation of DNA sequences from blastocysts and fetuses

DNA was isolated from blastocysts following lysis of individual blastocysts in 10 µL of buffer (QE09050, Biosearch Technologies, UK). Blastocysts were vortexed, centrifuged, incubated for 6 min at 65 °C and 98 °C for 2 min prior to storage at − 20 °C. Two rounds of PCR were performed using GoTaq Green Master Mix (Promega Biosciences, Madison, WI, USA) and primers designed to flank each target *AR* sequence (Table 3). The first reaction typically used 9.2 μL of lysed embryo (but as little as 5 µL), and the second reaction used 5 μL of PCR product from the first reaction. The PCR conditions were 5 min at 95 °C, followed by 35 cycles of 45 s at 95 °C, 45 s at annealing temperature, and 1 min at 72 °C. After 35 cycles, reactions concluded with 7 min at 72 °C.

**Table 3:** PCR primers used to amplify mRNA transcripts.

Primer name	Product size (bp)	Sequence ID	Efficiency (R^2^)	Sequence (5′→3′)^a^
AR_108F AR_108R	108	KU705631.1	107% (0.99)	TACCTGTGTGCCAGCAGAAAT AGCTCCCAGTGTCATCCCT
GAPDH_F GAPDH_R	104	KJ786424.1	92% (0.99)	GGTGAAGGTCGGAGTGAACG TGAAGGGGTCATTGATGGCG
U2_F U2_R	105	ENSSSCG000 00042922	105% (0.96)	GCCTTTTGGCTAAGATCAAGTGT GGCTTTCTATACCTTCCACAATCT
CYP17A_F CYP17A_R	132	AH003260.2	109% (0.99)	GTGCTCTTGGTTTTCTTCTTGCT ACGTCTGGGTAGGAATGGC
AR_RNAseqF AR_RNAseqR	357	KU705631.1		GTGTGGAGGCATTGGAGCAT AGAGGAAAGTTGTAGTAGTCGTTC

^a^Primer concentrations were 75 nM for *AR*, 50 nM for *GAPDH,* 200 nm for U2, and 400 nM for *CYP17A*. The 357 bp product was a product of RTPCR used for sequencing while other primers were used in qPCR reactions.

Tail samples were removed from recovered fetuses and DNA was extracted using Qiagen’s DNeasy Blood and Tissue Kit (#69504). DNA concentration was determined by nanodrop (Thermo Scientific NanoDrop™ 2000). A single round of PCR was performed using extracted fetus DNA, GoTaq Green Master Mix (Promega Biosciences), and specific primers designed to flank each target *AR* sequence (Table 3) using PCR conditions described above for blastocysts.

PCR products were loaded onto a 0.8% agarose gel (1X TBE buffer) and separated by gel electrophoresis. Bands were excised under UV transillumination using a clean razor blade. DNA was extracted and purified using the QIAquick Gel Extraction Kit (Qiagen #28704). Extracted DNA was submitted to Genewiz (South San Francisco, CA) for Sanger sequencing of *AR* in all potentially edited fetuses. Sequences were aligned to the reference sequence using SnapGene^®^ (from Insightful Science; available at snapgene.com) and CRISP-ID^41^ software.

### XY sequencing of edited blastocysts and fetuses

For each blastocyst, 5 μL of lysate was used for the XY PCR reaction and the remaining 5 μL was used for the initial *AR* PCR described above. Nested PCR was performed using GoTaq Green Master Mix (Promega Biosciences) with primers designed to amplify regions of the X and Y chromosome^42,43^ and an additional reverse primer for the Y chromosome, CTCTGTGCCTCCTCGAAGAAT, that makes a 154 bp product. PCR conditions were 5 min at 95 °C, followed by 30 cycles of 30 s at 95 °C, 30 s at 55 °C, and 30 s at 72 °C, and concluded with 7 min at 72 °C. PCR product from the first reaction (5 μL) was used for the second reaction. PCR products were visualized on a 2% agarose gel (1X TBE buffer) with SYBR™ Safe DNA gel stain (Invitrogen™) to determine sex. Sex of each fetus was similarly evaluated but sufficient DNA was available that a single round of PCR was adequate.

### RNA isolation, PCR and qRT-PCR

After pulverization of fetal tissue in liquid nitrogen using a mortar and pestle, total RNA was extracted using Trizol^®^ reagent (1 mL, Invitrogen, Waltham, MA, USA) as per manufacturer directions. The RNA pellet was washed a second time with 75% ethanol. After air drying and resuspension in RNAse-free water, the RNA was briefly incubated at 55 °C prior to determination of concentration with a NanoDrop 2000 spectrophotometer (Thermo Scientific). The A260/A280 ratio was used to evaluate RNA purity, and RNA was stored at − 80 °C. RNA samples were treated with DNase (M6101, Promega, Madison, Wisconsin, USA) to remove genomic DNA. First strand cDNA synthesis was completed with the Thermo Scientific RevertAid first strand cDNA synthesis kit (K1621).

Primers to detect *CYP17A1 and U2.35* mRNA expression were designed using Primer Blast^40^ (Table 3) and dimer formation and secondary structure of primers analyzed with Net Primer (Premier Biosoft, Palo Alto, CA, USA). Correct product size was confirmed by agarose gel electrophoresis and melt curves confirmed synthesis of a single product. *GAPDH* was used as the reference gene. Real-time quantitative PCR reactions were set up in triplicate using PowerUp SYBR Green Master Mix (#A25742, Applied Biosystems, Waltham MA, USA) and an Applied Biosystems QuantStudio™ 3 PCR machine. Final reaction volume was 20 µL containing cDNA synthesized from 5 ng RNA and amplification temperature program was 95 °C for 20 s, followed by 40 cycles of 95 °C for 3 s and 60 °C for 30 s; after amplification, the melt curve was initiated. Standard curves were generated for all primer pairs (Table 3); efficiencies were between 90 and 110%.

### Presence of AR transcript in head and body of control fetuses

RNA was isolated separately from the heads and bodies of control fetuses collected 19 days after fertilization and cDNA synthesized as described above. The presence of *AR* transcripts in both heads and bodies of male and female fetuses was confirmed by traditional PCR followed by agarose gel electrophoresis. Similarity in relative transcript levels was evaluated at this age using qRT-PCR and the same *AR* primers. An additional set of primers targeting the *AR* transcript (357 bp product) was designed to allow sequencing of this transcript to confirm identity (Table 3).

### Statistics

Differences in sex ratio from the expected 50:50 ratio were evaluated by Chi square analysis. Differences in gene expression detected by qPCR (ΔCt) were analyzed by analysis of variance (aov) using R statistical programs^44^ after checking for homogeneity of variance and normality with Bartlett, Levene, and Shapiro tests.

### Supplementary Information

Supplementary Figure 1.

## Data Availability

Most data generated during this study are included in the published article. An additional dataset including altered DNA sequences in *AR*-edited blastocysts generated in this study is available at DRYAD repository, 10.25338/B88K97.
