# Supplementary figures and images for: Gender disparity in survival of early porcine fetuses due to altered androgen receptor or associated U2 spliceosome component
Source: Sci Rep. 2023 Sep 12;13:15072. doi: 10.1038/s41598-023-41665-6 (PMC10497509; doi:10.1038/s41598-023-41665-6)

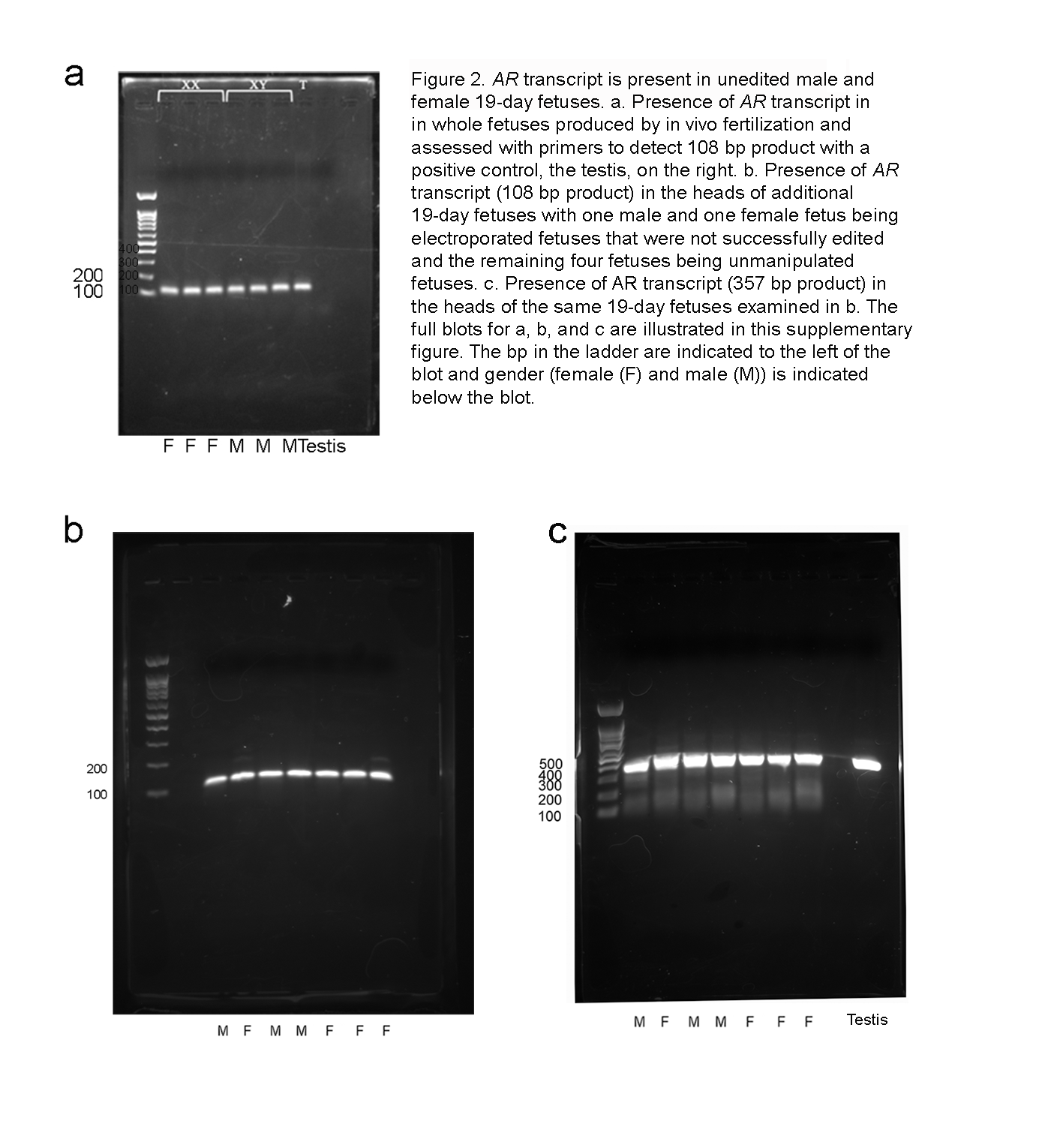

Supplement: Supplementary file 1 — Supplementary Figure 1. [file 41598_2023_41665_MOESM1_ESM.tif]
